# Analysis of the effect of digital hospital efforts on paper savings in inpatient procedures and on the duration of nursing care services

**DOI:** 10.3389/fdgth.2024.1367149

**Published:** 2024-06-03

**Authors:** Esra Volkan, İlker Köse, Sinem Cece, Özge Elmas

**Affiliations:** ^1^ Department of Health Management, Istanbul Medipol University, Istanbul, Türkiye; ^2^ Department of Computer Science, Alanya University, Antalya, Türkiye; ^3^ Department of Management Information Systems, Ankara Medipol University, Ankara, Türkiye; ^4^ Department of Technology Transfer Office, Alanya University, Antalya, Türkiye

**Keywords:** healthcare costs, health informatics, medical record, nursing care, time study

## Abstract

**Background:**

This study has two primary objectives. Firstly, it aims to measure the time savings achieved through the digitization of paper forms filled out by nurses in the inpatient care process. Secondly, it seeks to reveal the financial savings resulting from reduced paper consumption due to the digitalization. The Health Information Management System Society (HIMSS)—Electronic Medical Record Adaption Model (EMRAM), which makes stage-based (0–7) evaluations, serves as a tool to measure the rate of technology utilization in public hospitals in Turkey. The study is based on the HIMSS EMRAM criteria for 2018. Bahçelievler State Hospital, a public hospital in Turkey, was chosen as the research facility. In 2017, it was accredited as Stage 6 with HIMSS EMRAM. However, not all its wards have been digitalized. Initially, pilot selected wards were digitized. Therefore, digital and non-digital wards serve together. In this context, 4 wards were randomly selected and time, paper and toner savings before and after digitalization were measured.

**Method:**

A table was created in Microsoft Excel,listing the forms used by nurses in inpatient care and the time required to fill them out.The time spent for filling paper-based forms and digital-based forms was measured in randomly selected wards.

**Result:**

The analysis showed that digital forms saved more time, paper and toner. For example, filling out the patient history form took 45 min when using paper, compared to 12 min in digital environment. Approximately 27% time savings are achieved only for the patient history form. The total time savings delivered by digitalization for 1,153 inpatients during the year were found as 117 care days, and the savings on total paper consumption was 41.289 pages. For 1,153 inpatients throughout the year, the total time savings from digitalization was 117 care days and the total paper consumption savings was 41,289 pages. In addition, in 4 wards with a total bed capacity of 25, annual paper savings of $1,705.86 and toner savings of $283,736 were achieved.

**Discussion:**

This study reveals the benefits of digitalisation in hospitals for nurses. It saves the time that nurses allocate for filling out paper forms with digitalised forms. Thus, it is a good practice example in terms of using the time allocated for form filling for patient care.When we extend this study to Turkey in general, it can be considered that the time savings achieved by nurses by digitizing inpatient forms varies between 10.8% and 13%. The number of nurses working in public hospitals in Turkey is approximately 160,000. Assuming that 60% of the nurses work in the inpatient ward, it is understood that the annual savings achieved by digitizing the forms corresponds to a range of 398–559 nursing hours.

## Introduction

Nursing is a dynamic profession that has transcended its traditional boundaries and continues to develop unique theories. Nursing/midwifery is a profession that directly engager with and serve individuals. The field of health differs from other business lines because individuals living under intense stress, namely patients, are served, and personnel working in this field often confront stressful situations (1). The job descriptions of nurses encompass a variety of responsibilities, includingcollecting patient data, assessing patient needs,, performing nursing diagnosis, developing and implementingcare plans, monitoringmedication administration, training patients and their relatives, and arranging patient transfers when necessary (2).

Considering that all these processes are carried out on paper, and there are often not adequate nurses, it has been observed that nurses spend a pretty long time for each patient record they make in hardcopies. This being the case, the use of electronic health records in nursing processes has come to the fore, and it is considered that hospital information and management systems will help increase the care time to be allocated to the patient by reducing the time allocated to stationery operations (3).

It has been stated that if the nursing forms and work processes are fulfilled through Hospital Information Management System (HIMS), this will result in enhanced health care quality, decreased paperwork, reduced paper consumption, and increased nursing performance and satisfaction induced by reduced workload (4). The willingness of nursing staff to embrace digital technology is generally high. For example, the integration of medical devices with the hospital information management systems accelerates the work of nurses. Studies areseen that the efficiency and motivation among nurses increase significantly with digitalization (5).

This study aims to compare the time nurses spend in inpatient services when the records (filled forms) of health care are kept in the form of hardcopies and electronic media and to measure the change in paper consumption when electronic systems are used.

### Effect of electronic systems on health service quality, nurse performance, and satisfaction

A wide variety of high-level information is produced in health care delivery and treatment processes. Therefore, it becomes necessary to use information technology-supported systems to record this information, use it in the treatment process, facilitate retrospective access to information and manage the treatment process in a quality manner. The systems used by health institutions for this purpose are called Hospital Information Management Systems (HIMS). A valuable, and well-designed HIMS is highly correlated with health care quality (6).

It is seen that hospitals which are using information systems have come a long way in terms of quality. It is observed that the quality of health services has increased in organizations that keep up with the changing health technologies, make investments for this purpose and actively use the technologies in the field. In 2009, Kilinc investigated the effect of information technologies on improving the service quality. Based on the questionnaire results applied to 165 patients from 4 different hospitals in Konya province, a strong relationship was determined between the quality of the service provided and the use of technology (7).

Another study on this subject was conducted by Banet et al., where nurses were caused to use HIMS for 12 months. The measurement was conducted using a questionnaire with 55 nurses before and after 1 year. It was observed that the time spent by the nurses for recording decreases, and the time they allocate the patients' care increases since nurses record all treatment and care processes electronically and avoid using paper. In addition, it was determined that the performance of nurses increased due to the decrease in the time allocated to nursing work and procedures (8).

The results of the HIMS usability questionnaire for nurses were analyzed in the study conducted by Yılmaz and Demirkan, which evaluated the effect of electronic systems on nurse satisfaction and assessed the usability of hospital information and management systems. Nurses were asked to present their opinions about HIMS, practical usability, learnability, assistance, safety, customization, design, satisfaction, and ease of use. In this study, the concept of satisfaction was defined as the satisfaction of the healthcare personnel using the HIMS with the software, their feeling of content with the speed and accuracy of the software, and the assistance provided by the software to allow them to do their jobs faster (9).

Nurses are direct users of HIMS. The usability of the HIMS interface is of great importance for nurses due to the complex structure of nursing work and operations. It is predicted that a HIMS that warns and helps nurses when faced with adverse circumstances, is not obstructive but suggestive in decision-making, organizes nursing workflows, and has a user-friendly interface will enhance satisfaction from nursing services (10). The study conducted by Saluvan, which supports the conclusion that the use of information technologies is required to improve quality, concluded that the use of information technologies could positively affect the quality of care since health services have a complex structure (11).

The purpose of usability is to produce products that are compatible with the information and usage habits of the user. To increase the usability of HIMS, the expectations of users from the product and the product itself should be designed to meet the user's needs. Thanks to the information systems designed considering the information and usage habits of the user, the effectiveness, efficiency, and user satisfaction delivered by the product increase (12).

A study by Yorgancioglu et al., investigated the effect of innovation studies on health service delivery. A questionnaire was applied to a total of 143 patients treated at Eskisehir State Hospital. Based on the research results, the technological system and hardware infrastructure in the hospital increase the quality of the health service offered. Additionally, this induces a positive effect on patients. At the same time, it was determined that some elements, such as electronic records, physician and nurse electronic documentation, use of technology in clinical decision-making, and electronic medication management and administration, affect the quality perception among patients positively (13).

In a study conducted by Nokay et al., which deals with the effect of electronic systems on nurse performance, a questionnaire was applied to 307 healthcare professionals to determine the effect of electronic systems on institutional performance. Based on the survey study results, the operating speed and performance of healthcare personnel using electronic systems are elevated (14). Interventions used to facilitate nurse education and training on electronic health records are also important. Inadequate education and training can threaten the adoption of electronic health records and negatively affect the quality of nursing documentation. Therefore, as well as the digitization of nursing forms, nurses need to be taught how to complete these forms (15). With the digitalization of nursing forms, it was observed that the transition from paper-based documentation to computerized documentation was easier, nurses using the electronic nursing documentation system received more approvals, and complaints about its content in the practice environment were minimized (16).

Another study suggesting that digitalisation in healthcare institutions increases patient care and safety was conducted by Vida et al. When a digitalisation strategy is determined for healthcare institutions, it has been observed that its effect on patient safety and care is positive (17). A study conducted in Australia in 2017 investigated the use of digital technology in healthcare settings. Interviews were conducted with nurses and the barriers to nurses' access to digital technology were discussed. It was observed that nurses could not use technology in patient care because they did not know how to access technology. In interviews with nurses, it was concluded that the use of technology will benefit health care services (18). In the study conducted by Krick et al., the existence, use and benefits of digital technologies in nursing care were discussed with a literature review. In particular, it was observed that studies on the use of technology in the field of nursing are quite rare (19).

This study proved the benefits of digitalizing nursing forms in the literature. It is seen that questionnaire and time study are used as measurement methods in the studies. In this study, time study was preferred as a method. With the survey method, the satisfaction of nurses about solid and digital system applications can be analysed comparatively. This study determines the effect of electronic filling of nursing forms instead of paper in inpatient procedures within the scope of digital hospital studies on the duration of nursing care services and measures the cost-effectiveness of filling out the forms electronically on paper consumption.

## Methods

This study is based on the data of 1,153 patients hospitalized in the Internal Medicine, Infection, Chest Diseases, and Neurology ward of Bahcelievler State Hospital between January 2023 and January 2024. Two types of data were collected within the scope of the study. The first type of data recordedthe time nurses spent filling out paper forms during inpatient services. The second type of data capturedthe time nurses spent filling out the same forms using the Hospital Information Management System (HIMS). Personal Data Protection Law No. 6698 was taken into consideration during the data acquisition, and the distinctive identity data of the patient was completely removed from these data before they were used. Also, informed consent and assent were obtained from the patients. Patients were informed about the scope of the study.

This study consists of two separate analyses. In the first analysis, the researcher used an observation method and compared the time spent by nurses in filling out paper forms vs. electronic forms in the wards under study. A Microsoft Excel file was created to collect these data. The Excel file consisted of the following columns:

• Patient name
• Ward of admission
• Names of nursing forms
• Time spent on filling out paper forms
• Time spent on filling out electronic forms

First, the time spent by nurses on filling out paper forms was recorded by the researcher using a stopwatch. Then, the time spent by nurses on filling out electronic forms while entering data into the Hospital Information System (HIS) was calculated. Thus, data were collected to compare both situations. It should be noted that variations in forms used based on the ward of admission were not considered in this study. Only forms used universally across all patients were taken into account. The time required for nurses to fill out the forms (paper/electronic) was determined based on the number of days patients were admitted, and the total time spent by nurses in filling out forms was calculated accordingly.

In the second analysis, the potential paper savings from digitizing paper forms and active use of the HIS were calculated. Another Microsoft Excel file was created to collect these data. The Excel file consisted of the following columns:

• Form name
• Number of pages per form
• Frequency of form usage

*Some forms were filled out only once, while others were repeated throughout the duration of the patient's admission. Therefore, calculations were made taking into account the number of days the patient was admitted and the number of pages for forms that were repeated*.

The information regarding the number of days patients were admitted and the number of pages for forms was provided by the hospital management. Subsequently, the total paper consumption was calculated by multiplying the number of days each patient was admitted by the number of pages for forms used during that period, considering eight nursing forms included in the study.

The forms filled out by the nurses contain the following patient information:

• Patient Admission Number
• Date of Admission
• Hospitalization Release Date
• Number of Hospitalized Days
• Type of Discharge
• Details of the Ward Where the Patient Is Hospitalized

The forms included in the research were selected from those filled out by the service nurses for each patient in the inpatient wards.Nurses using the digital system and nurses using the paper-based system were randomly selected. Because paper and digital forms used for inpatients throughout the hospital are the same. The completed forms for a particular age group or disease were excluded (e.g., geriatric forms, diabetes forms).

1. **Forms included in the scope of the study**
  (1) Nursing History Form
  (2) (Itaki) Adult Patient Fall Evaluation and Follow-up Form
  (3) Nurse Observation Form
  (4) Form Nrs2002
  (5) Pressure Sore Risk Assessment Form (Norton Scale)
  (6) Das (Behavioural) Pain Rating Scale
  (7) Patient and Companion Training Form
  (8) Nurse Follow-up Form (Care Plan)

In this study, the researcher used the observation method and recorded the paper-based form-filling times of the nurses by observing them one-on-one on-site. A table was created in Microsoft Excel for this calculation. The names of the patients, their wards, the names of the forms filled in and the time of filling in the forms were included in this table. Nurses' form filling times were monitored and calculated with a stopwatch. The digital-based form filling times of the nurses were calculated through the Hospital Information Management System. Thus, it was possible to make comparisons. In this study, it was not taken into account that the forms used varied according to the ward in which the patient was hospitalised. Printed forms used for all patients were taken into consideration. For inpatients, the time required to fill in the forms used by the nurses (according to the electronic/paper form) was determined. Basing the patients' hospitalization period, the nurses' total time spent filling out the forms was calculated. Another data collected within the scope of this study is the paper savings achieved when electronic-based forms are used instead of paper-based forms. For this purpose, the number of patients hospitalized in the selected wards and the number of days of hospital stay were determined. Then, the total paper consumption was calculated by multiplying the number of hospitalization days for each patient and the number of form pages used over the eight nursing forms used in inpatient services.

### Ethical considerations

Ethical approval was taken from the local ethics committee on 25/07/2018 with number 430. The approval was received from İstanbul Medipol University Health Science Ethical Committee.

## Results

### Time analysis

As seen in Table 1, the fill-out times and time differences for the nursing forms were filled out in digital media, and hardcopies were recorded starting from the patient's admission to the ward. The time spent on the forms during the observation was recorded in seconds. The time difference between digital and paper forms filled out by nurse A for one patient during the year was 13.7 min. Similarly, this value was calculated as 11.67 min for nurse B, 11.58 min for nurse C, and 11.55 min for nurse D.

**Table 1:** Comparison of time spent for a patient.

	A Nurse			B Nurse			C Nurse			D Nurse		
	Total time for digital forms	Total time for hardcopy forms	Total time difference	Total time for digital forms	Total time for hardcopy forms	Total time difference	Total time for digital forms	Total time for hardcopy forms	Total time difference	Total time for digital forms	Total time for hardcopy forms	Total time difference
Total time spent for forms filled out during hospitalization (s)	558	1.380	**822**	410	1.110	**700**	498	1,193	**695**	462	1,155	**693**
Total time spent for forms filled out during hospitalization (min)	9.3	23	**13.7**	6.83	18.5	**11.67**	8.3	19.88	**11.58**	7.7	19.25	**11.55**

Bold values show total difference values.

Table 2 shows the nurses' time on the forms for each patient hospitalized during the year. The nursing history form is filled out only once during admission to hospitalization. Other forms are the forms that are used repetitively every day during the hospitalization of the patient. The nurse observation form includes vital sign measurements repeated three times a day. The recurrence time was also considered when calculating the times spent to record the nursing observation form on paper and digitally, and the time spent for the recording process was calculated by multiplication by 3.

**Table 2:** Comparison of time spent by nursing forms in seconds.

Reviewed forms	A Nurse		B Nurse		C Nurse		D Nurse		Average	
	Time for hardcopy forms (s)	Time for digital forms (s)	Time for hardcopy forms (s)	Time for digital forms (s)	Time for hardcopy forms (s)	Time for digital forms (s)	Time for hardcopy forms	Time for digital forms (s)	H.copy form (s)	Digital form (s)
Nursing services patient history form (The patient's account of their medical history)	400	225	280	120	328	163	310	160	330	167
(Itaki) adult patient fall evaluation and follow-up form	130	45	90	35	110	40	95	37	106	40
Pressure sore risk measurement form (Norton scale)	65	45	33	15	45	25	50	32	48	30
das (behavioural) pain assessment scale	50	20	30	10	45	17	36	17	40	16
Nurse observation form	370	85	356	93	325	76	336	65	346	80
Nursing process care plan	180	75	162	70	155	67	166	63	166	68
Patient and companion training form	90	38	75	27	95	60	82	44	85	42
NRS2002 form	95	55	4	40	92	50	80	44	68	48
Total	1,380	558	1,110	410	1,193	498	1,155	462	1,210	482

In Table 3, the savings on time resulting from the conversion of nursing forms of 1,153 hospitalized patients from hardcopy forms to digital forms are analyzed by nurses. For example, the time difference for nurse A to fill out the the patient's account of their medical history form in hardcopy and digital forms is 175 s, as given in the previous table. Considering that the patient's account of their medical history form is filled out only once for each patient, the savings on time provided by Nurse A were multiplied by the total number of inpatients, and the total time savings during the year was determined as 201,775 s.

(1) 400–225 = 175 (Time difference when filling out hardcopy forms and digital forms)
(2) 175 × 1,153 = 201,775 s (Time difference multiplied by the total number of inpatients)
(3) 201,175/60 = 336 min (Converted from seconds to minutes)
(4) 336/60 = 56 h (Converted from minutes to hours)
(5) 56/8 = 7 days (Total working hours were determined as 8 h and regarded as 1 day. The total day savings were determined by dividing the total hours by 8)

**Table 3:** Calculation chart for 1-year time savings.

		Nurse A	Nurse B	Nurse C	Nurse D	Average
Nursing services patient history form (The patient's account of their medical history)	Time savings (s)	201,775	184,480	190,245	172,950	
	Time savings (min)	336	307	317	288	
	Time savings (h)	56	51	52	48	
	Time savings (day)	7	6.37	6.5	6	**6.5**
(Itaki) adult patient fall Evaluation and follow-up form Pressure sore risk measurement form (Norton scale) Das (behavioural) pain assessment scale Nurse observation form Nursing process care plan Patient and companion training form NRS2002 form	Time savings (s)	3,603.143	3,007,.60	2,951.570	3,023.967	
	Time savings (min)	60,052	50,121	49,192	50,399	
	Time savings (h)	1,000	835	819	839	
	Time saving (day)	125	104,375	102,375	104,875	**110**

Bold values show average of time savings (day) for nurses forms.

The same table indicates the savings on time achieved by switching the repetitive nursing forms used during the hospitalization from hardcopy to digital. For example, the times for completion of 7 forms mentioned above in hardcopy form and digital form by Nurse A are given in the previous table. Considering that other forms are repetitively used every day during the hospitalization, the total time savings achieved by nurse A during the year were multiplied by the total number of care days, which was found as 3,603.143 s.

(1) 980–333 = 647 (Time difference for seven forms when filling out in hardcopy and digital formats)
(2) 647 × 5,569 = 3,603.143 s (Time difference multiplied by the total number of care days)
(3) 3,603,143/60 = 60,052 (Converted from seconds to minutes)
(4) 60,052/60 = 1,000 (Converted from minutes to hours)

### Paper consumption analysis

The patient's account of their medical history form given in Table 4, which is filled out during the admission of the patient to any of the Internal Medicine, Infection, Chest Diseases, and Neurology wards, consists of 2 pages. The seven forms, which are utilized for daily follow-up after admission and repetitively used at certain intervals, consist of 7 pages in total.

**Table 4:** Number of forms used and number of their pages.

	Forms filled out only once during hospitalization	Forms used repetitively every day
Number of forms	1	7
Number of pages in a form	2	7

### Total paper consumption measurement

Table 5 gives a total of 5,569 days of care provided for 1,153 hospitalized patients between January 2023 and January 2024. The number of pages in the nursing the patient's account of their medical history form, which was filled out and was not repetitively used during hospitalization, was found to be 2,306 pages when multiplied by the total number of care days. The total number of pages of the seven forms, which were repetitively used daily during hospitalization, was found to be 38,983 pages when multiplied by the number of days of care. Additionally, it was determined that the total number of papers consumed for 1 year was 41,289 for 1,153 patients.

**Table 5:** General analysis of the study.

Number of care days	Number of pages in a form filled out only once during hospitalization	Number of pages in forms used repetitively every day	Overall total
5,569	2,306	38,983	**41,289**
Total number of pages in forms	Number of one ream of A4-size paper	Total number of A4 packs consumed	** **
41,289	500	82.57	** **
Total number of pages in forms	Number of usable papers per pack of toner	Total number of toner packs used	** **
41,289	1,600	25.80	** **
Total number of pages in forms	Number of usable papers per pack of toner	Total number of toner packs used	
41,289	1,600	25.80	** **
Total number of pages in forms	Price of a A4 box (2,500 pages) in $	Total paper cost	** **
41,289	21.06$	1,705, 86$	** **
Total number of pages in forms	Price of a toner (1,600 p. black) in $	Total toner cost	** **
41,289	103.07$	2,837,36$	** **

Bold value shows overall total value for 3 columns.

According to the Central Bank, the average dollar exchange rate on a level basis for 2023 is 23.74.

### Total number of reams consumed

Based on Table 5, it is provided that the total amount of reams (each ream contains 500 pages) consumed for 1 year is 82.57 for 1,153 patients.

### Total number of toners consumed

Table 5 shows that 25.80 packs of toners were used in total, considering that one pack of toner can be used for 1,600 pages, and the total number of pages is 41,289.

### Paper consumption cost

The information obtained from technology stores determined the average price of a 2,500-page A4 Paper box as 21,06$. Table 5 shows that the total annual paper cost for 1,153 patients is 1,705.86$.

### Total cost of toners consumed

Based on the information obtained from technology stores, the cost of a pack of toner, which can be used for 1,600 pages, is 103.07$. Table 5 shows that the total annual cost of toners for 1,153 patients is 2,837.36$.

### Turkey-scale projection

Considering the 90% bed occupancy rate in Bahcelievler State Hospital, it is understood that 22.5 of 25 beds in 4 wards are occupied at all times. In line with the study conducted with four nurses, it is seen that one nurse provides continuous care for 5.5 beds. Given the time each nurse spends for the forms and the time differences, we can determine how much of the total working time is allocated to documentation. The example given in Table 6 shows that nurse A spent 18 min on the hardcopy forms that she filled out for one patient, totaling 99 min for 5.5 patients. It was found that nurse A spent 20% of her 8-h shift filling out hardcopy forms. After digitalization, we see that nurse A spent 6.18 min on the digital forms she filled out for one patient, totaling 34 min for 5.5 patients. It was determined that nurse B spent 7% of her 8-h shift filling out a digital form. In line with all this information, it was established that nurse A saved 13% on time spent filling out forms after digitalization. When making the same calculations for other nurses, it was determined that nurse B saved 11% on the same time, followed by nurse C with 11% and nurse D with 10.8%.

**Table 6:** Rate of total working hours saved based on nurses after digitization.

** **	Rate of time spent on hardcopy forms	Rate of time spent on digital forms	Rate of total working hours saved
Nurse A	20%	7%	13%
Nurse B	16%	5%	11%
Nurse C	17%	6%	11%
Nurse D	17%	6.2%	10.8%

Since the the patient's account of their medical history form is a form that is filled out only once, the time saved by a nurse while filling out each the patient's account of their medical history form is divided by the total hospitalization days after calculation. The subsequent value found was summed up with the time savings delivered for other forms. Thus, the repetition of the time spent on the the patient's account of their medical history form was avoided.

After digitalization, it is seen that the total rate of savings in an 8-h working period ranges between 10.8% and 13%.

Considering the cost of paper consumption derived from our study for 25 beds, it is possible to note that a paper cost savings of approximately 533,287.18$ per year will be achieved in a service offering with 135,340 beds if we build a projection throughout Turkey.

## Discussion

Informatics skills are increasingly becoming a necessity for nurses. Nurses will use technology more frequently in the future and must be adequately prepared to document patient care and analyze data. Therefore, they will be expected to improve their informatics skills throughout their ongoing education. In a study conducted at the California School of Nursing, a simulation study was conducted with third- and fourth-year nursing students on using electronic health records. In this study involving 38 nursing students, the students were given simulated training on using electronic health records for two terms in total. At the end of the study, the students stated that the use of electronic health records in hospitals establishes a bond of trust between the patient and the nurse and added that it is easier to access the patient's history by recording the data and responding to the patient inaccurately is avoided with the help of the clinical warnings created (20).

A study conducted in the US investigated whether electronic health records are effective in the varying health conditions of patients in an intensive care unit. During face-to-face interviews with nurses, nurses were asked how they used the electronic health record to assess patients' conditions. In the study involving 18 nurses, 47% of the nurses stated that they were able to regularly monitor the patient's vital signs through the electronic health record, and thus, they could make healthier and stronger decisions during the care process. On the other hand, 32% of the respondents find the use of clinical templates useful and think that it accelerates data recording and access to data (21). A similar study by Pabst et al., measured the effect of electronic systems on nursing documentation time. According to the study, nurses using electronic systems could reduce the time they allocate to documentation and lengthen the time they allocate to the patient (22).

The literature includes few studies that measure the effect of using electronic health records on time. In a study by Poissant et al., the effect of using electronic health records on the time efficiency of physicians and nurses was investigated. Based on the study, which examined and compiled a total of 23 publications, it was observed that using electronic health records in nursing work processes allows nurses to save time by 23.5% (23).

In parallel to our study, a study conducted by Banner et al., observed before and after the electronic use of nursing forms. The study's results established that the time spenton nursing documentation decreased by 12% after digitalization (24). The master's thesis study conducted in 2019 measured the annual time and paper savings achieved after digitalization in intensive care units. Based on the study conducted with two nurses, after the digitalization of the daily forms in intensive care units, it was determined that nurse X saved 48 min per day and nurse Y 65.50 min per day. It was determined that the annual time savings achieved were 248.20 care days for nurse Y and 181.44 care days for nurse X (25). In this study, the time spent by nurses for care in partial digital (ward using both paper and digital forms) and digital clinic was compared. It was observed that 129.1 min were spent for indirect care practices in the full digital clinic and 404.4 min in the digital clinic. In addition, in the partially digital ward, 281.5 min were wasted due to medication preparation in the treatment room, preparation/use of paper-based documents, double entry of some procedures, nurses collecting data from both digital and paper systems, and additional e-order entry for medications prescribed by doctors. This study supports our study in terms of proving that when nurses use paper forms, the time allocated to patient care is shortened (26).

Similar results were derived in our study based on a total of 5,569 care days of 1,153 patients hospitalized in the Internal Medicine, Infection, Chest Diseases, and Neurology wards at Istanbul Bahcelievler State Hospital between January 2023 and January 2024. In the process that started with the patient admission to the ward, the routine procedures fulfilled by the nurses were followed, and the forms used as a standard for each patient were reviewed individually. The forms filled out in hardcopy format before the digitalization process were also reviewed in the electronic environment after digitalization. The electronic and manual fill-out times of the forms were observed upon the researcher's involvement in the environment of the researched subject. Based on this observation, the times required to fill out the forms electronically and manually were determined. Upon the observational measurement conducted with four ward nurses, the savings on time delivered for 1 year regarding the given forms were calculated as 117 working days (1 day was calculated as 8 h). The four wards we examined have a total of 25 beds, with four nurses working. Likewise, the results of our study based on 5,569 care days of 1,153 patients demonstrated that 1.76 nurses were saved after digitalization.

For the Turkey-scale projection; it is understood that the annual savings in Turkey correspond to a nursing time ranging between 398 and 559, based on the projection made in consideration of the minimum 10.8% and maximum 13% savings indicated by our study. When reviewing similar studies in the literature, the effect of electronic health records on nursing documentation time has been emphasized, but the paper savings achieved upon the digitalization of forms have not been measured. Additionally, our study measured the paper and toner savings achieved due to the digitalization of nursing forms. Based on our study carried out in 4 wards with a total bed capacity of 25, the paper savings achieved by switching eight nursing forms filled out for 1,153 hospitalized patients to digital format during the year are 41,289 pages. In addition, the savings on toner costs amount to $ 2,837.36. Based on a study conducted in the intensive care unit in 2019, which measured the paper savings achieved after digitalisation, papers worth $707.29 and toners worth $4,977.02 were saved annually in a 22-bed intensive care unit, as similarly indicated by our study (25).

## Conclusion

Patient loyalty can be evaluated as a continuation of this study. Satisfaction with the patient care process carried out with paper forms and satisfaction with the patient care process carried out with digital forms can be compared. Thus, a new contribution to the literature will be provided. It is a fact that data that are not recorded and analysed electronically do not contribute to any academic research. In today's conditions, producing academic studies from data on paper takes a lot of time and requires labour force. Information kept on paper cannot become data and cannot be analysed. Considering that a total of 41,289 pages of paper is consumed in 1 year for 1,153 hospitalised patients, retrospective examination and analysis of these documents for academic studies is a heavy burden. For healthcare organisations, digitalisation is very important not only in terms of patient care but also financially. The use of paper creates a financial burden when evaluated on a country basis. Digitalisation is a measurement tool especially for the health ministries and policy makers of countries. With digitalisation, access to patient data becomes easier, the time allocated to the health care process of patients increases and the potential of patients to access health outcomes increases.

The study covers a period of only 1 year. It is not thought that the result may change when spread over a longer period. Because currently, hospitals in Turkey still use a dual system. While some hospitals have completely switched to the digital system, some hospitals use both the raw digital system and the paper system together. The forms used in hospitals in Turkey are determined by the Ministry of Health. Therefore, the same forms are used in public hospitals throughout the country. Therefore, it makes it easier to generalise across the country. However, a limitation of the study is the size of the hospital. Hospital size is determined depending on the number of beds. In such a case, the number of forms used in a hospital with a high number of beds will increase and financial calculations will be different. As a continuation of this study, the use of paper forms and digital forms according to hospital size can be discussed comparatively.

As a result, considering the potential benefits of digitalisation in healthcare institutions for all stakeholders, the concept of digital hospital should be expanded and all processes should be carried out electronically.

Future research could explore the long-term effects of digitalization on patient outcomes, healthcare costs, and overall quality of care. Additionally, comparative studies across different healthcare systems or countries could provide valuable insights into the scalability and generalizability of digital hospital initiatives.

## Data Availability

The raw data supporting the conclusions of this article will be made available by the authors, without undue reservation.

## References

[1] Yıldırım N. Erzurum İlinde 1. Basamak Sağlık Hizmetlerinde Görev Yapan Hemşire ve Ebelerin Tükenmişlik Düzeyleri. Atatürk Üniversitesi (2009).

[2] Kavaklı Ö Uzun Ş Arslan F. Yoğun Bakim Hemşirelerinin Profesyonel Davranişlarinin Belirlenmesi. Gülhane Tıp Dergisi. (2009) 51(6):168–73.

[3] Saluvan M. Sağlık Hizmetlerinin Kalitesini Iyileştirmede Bilgi Sistemlerinin Rolü. Ankara Sağlık Bilimleri Dergisi. (2013) 2:25–39. 10.1501/Asbd_0000000040

[4] Ozkul Ozel H Ozdemir Urkmez D Demiray S Cebeci Z. Hemşirelik bilişimi ve hastane bilgi yönetimi sistemi. Med J Okmeydani Train Res Hosp. (2014) 30(3):158–60. 10.5222/otd.2014.158

[5] Korte L Bohnet-Joschko S. Digitization in everyday nursing care: a vignette study in German hospitals. Int J Environ Res Public Health. (2022) 19(17):10775. 10.3390/ijerph191710775 36078491 PMC9518544

[6] Kimiyafar K Moradi G Sadooghi F Sarbaz M. Views of users towards the quality of hospital information system in training hospitals affiliated to Mashhad university of medical sciences-2006. Heal Inf Manag. (2006) 52:10.

[7] Kılınç C. Küreselleşme Sürecinde Teknoloji Yönetiminin ve Bilişim Teknolojilerinin Hizmet Kalitesini Artırmaya Etkisi ve Sağlık Sektöründe Bulunan Hastanelere Uygulanması. Selçuk Üniversitesi (2006).

[8] Banet GA Jeffe DB Williams JA Asaro PV. Effects of implementing computerized practitioner order entry and nursing documentation on nursing workflow in an emergency department. J Healthc Inf Manag. (2006) 20:45–54. 16669588

[9] Yılmaz M Demirkan AE. Hastane Yönetim ve Bilgi Sisteminin Kullanılabilirliğinin Değerlendirilmesi. Bilişim Teknolojileri Dergisi. (2012) 5(3):19–28.

[10] Rojas CL Seckman CA. The informatics nurse specialist role in electronic health record usability evaluation. Comput Inform Nurs. (2014) 32(5):214–20. 10.1097/CIN.0000000000000042 24473121

[11] Saluvan M. Sağlik Hizmetlerinin Kalitesi ile Hastane Bilgi Sistemleri Ilişkisi. Doktora tezi. Hacettepe Üniversitesi, Sosyal Bilimler Enstitüsü (2015). Available online at: http://www.openaccess.hacettepe.edu.tr:8080/xmlui/handle/11655/2478

[12] Yaldır A Taşer M. Hastane Bilgi Yönetim Sistemleri Için Olap Yöntemleri ile Karar Destek Modülü Tasarimi Ve Uygulamasi. Gazi Üniversitesi İktisadi ve İdari Bilimler Fakültesi Dergisi. (2016) 1:153–71.

[13] Yorgancioglu Tarcan G Karahan A Tarcan M. The effect of hospital innovations on perceived service quality: public hospital example. Online Acad J Inf Technol. (2018) 9(32):149–62. 10.5824/1309-1581.2018.2.009.x

[14] Nokay, İ Özaydın A. Effect of information technology services on hospital performance. Health Care Acad J. (2018) 5(2):226. 10.5455/sad.13-1525105454

[15] Ting J Garnett A Donelle L. Nursing education and training on electronic health record systems: an integrative review. Nurse Educ Pract. (2021) 55:103168. 10.1016/j.nepr.2021.103168 34411879

[16] Shafiee M Shanbehzadeh M Nassari Z Kazemi-Arpanahi H. Development and evaluation of an electronic nursing documentation system. BMC Nurs. (2022) 21(1):15. 10.1186/s12912-021-00790-1 35012513 PMC8744243

[17] Vida Z Vissi B Palicz T Lám J. Smart & safe—digitization strategy from a patient safety perspective. Orv Hetil. (2021) 162(47):1876–84. 10.1556/650.2021.32289 34801981

[18] Mather C Cummings E Gale F. Nurses as stakeholders in the adoption of mobile technology in Australian health care environments: interview study. JMIR Nurs. (2019) 2(1):e14279 10.2196/14279 34345771 PMC8279446

[19] Krick T Huter K Domhoff D Schmidt A Rothgang H Wolf-Ostermann K. Dijital Teknoloji ve Hemşirelik Bakimi: Resmi Olmayan ve Resmi Bakim Teknolojilerinin Kabulü, Etkililiği ve Verimliliği Çalişmalarina Ilişkin Kapsamli Bir Inceleme. BMC Health Serv Res. (2019) 19:400. 10.1186/s12913-019-4238-3 31221133 PMC6585079

[20] Mountaın C Redd R O’Leary-Kelly C Gıles K. Electronic medical record in the simulation hospital. Comput Inform Nurs. (2015) 33(4):166–71. 10.1097/CIN.0000000000000144 25887108

[21] Despins LA Wakefield BJ. The role of the electronic medical record in the intensive care unit “nurse’s” detection of patient deterioration. Comput Inform Nurs. (2018) 36(6):284–92. 10.1097/CIN.0000000000000431 29601339

[22] Pabst MK Scherubel JC Minnick AF. The impact of computerized documentation on “nurses” use of time. Comput Nurs*.* (n.d.) 14(1):25–30. Available online at: http://www.ncbi.nlm.nih.gov/pubmed/8605657 8605657

[23] Poissant L. The impact of electronic health records on time efficiency of physicians and nurses: a systematic review. J Am Med Inform Assoc. (2005) 12(5):505–16. 10.1197/jamia.M1700 15905487 PMC1205599

[24] Banner L Olney CM. Automated clinical documentation. Comput Inform Nurs. (2009) 27(2):75–81. 10.1097/NCN.0b013e318197287d 21685832

[25] Yilmaztürk N Kose İ Cece S. The effect of digitalization of nursing forms in ICUs on time and cost. BMC Nurs. (2023) 22(1):1–7. 10.1186/s12912-023-01333-6 37312143 PMC10262128

[26] Uzun LN Cerit B. Effect of digitalization on nursing practices using the lean approach. Clin Exp Health Sci. (2023) 13(3):450–9. 10.33808/clinexphealthsci.904203

